# New Insights Into Visual Word Recognition: Analyzing Error Distribution in Typical Readers

**DOI:** 10.5334/joc.441

**Published:** 2025-04-03

**Authors:** Fanny Grisetto, Clémence Roger, Gwendoline Mahé

**Affiliations:** 1 Univ. Lille, CNRS, UMR 9193 – SCALab - Sciences Cognitives et Sciences Affectives, F-59000 Lille, France; 2 Université de Reims Champagne-Ardenne, C2S, Reims, France

**Keywords:** error dynamics, visual word recognition, lexical decision task, conditional accuracy function

## Abstract

Previous studies have examined error dynamics to investigate the origins of incorrect lexical access. The comparison of correct and incorrect reaction times (RTs) and the use of Conditional Accuracy Functions (CAFs) in lexical decision tasks have led to inconclusive findings. The present study aimed to clarify these inconsistencies by integrating both methodological approaches across a larger dataset. Our results revealed a pattern of fast errors for pseudowords in both measures, with faster error trials compared to correct trials and a marked decrease in accuracy for the fastest trials. This pattern is discussed within diffusion models of visual word recognition and cognitive control which suggest that pseudoword errors are associated with uninhibited automatic lexical activation. Word errors appeared relatively insensitive to RTs, as no significant difference was found between correct and error RTs, and the CAF displayed a more uniform pattern, but yet not homogeneous. Indeed, a pattern of slow errors was observed for both words and pseudowords in the CAFs, with less accuracy in the slowest RTs. An exploratory analysis suggested that this pattern of slow errors in the word condition might be characteristic of poor reading skills. These aspects are discussed in regard to visual word recognition models that postulate several factors to explain the occurrence of slow errors. Taken together, this research provides a framework that could be used for identifying cognitive markers of reading difficulties. Future research could explore how factors like word frequency or reading skills influence error dynamics, potentially informing interventions targeting lexical retrieval deficits.

## 1. Introduction

Understanding the origins of reading difficulties, which affect a substantial portion of the population (OECD, 2019), is essential for developing effective reading interventions. Visual word recognition (VWR) is a major component of reading skills, and is thus largely targeted in reading difficulties studies. Research on VWR, primarily utilizing lexical decision tasks, has largely focused on sublexical and lexical components that contribute to accurate lexical access (e.g., word frequency in Brysbaert et al., 2018; first syllable frequency in Carreiras et al., 1993). However, a central, unresolved question in visual word recognition research concerns the origins of errors during VWR (i.e., why participants make “no” responses to words or “yes” responses to pseudowords). A first approach to answering “why” is to look at “when” errors are committed during lexical decision tasks, to provide some insights as to the timing of incorrect lexical access.

A first methodology to investigate error timing in the lexical decision task is to analyze the difference in reaction times (RTs) between correct responses and errors. Doing so, Horowitz-Kraus & Breztnitz (2011) reported slower RTs in error trials compared to those in correct trials. Interestingly, this difference was more pronounced in dyslexic readers compared to expert readers. For the authors, this lengthening of RTs in errors could arise from hesitation in expert readers, while in dyslexic readers, it could stem from unstable orthographic and/or phonological mechanisms. However, these interpretations should be considered with caution for two reasons. First, the duration of the stimuli display was extremely short in their study (i.e., 100 ms) which could have hindered decision-making processes (e.g., degradation of stimulus perception). Second, their analysis did not distinguish between word and pseudoword trials, for which differences in error timing have been observed by Ratcliff et al., (2004). In one of their experiments, the authors reported that RTs for errors were indeed faster than for correct responses when processing high-frequency words and pseudowords, whereas the opposite pattern was observed for words with low and very low frequency. However, these findings should be also taken with caution, as they were not replicated in all of their experiments. The failure to replicate may stem from the fact that comparing only mean RTs between correct and error trials may overshadow subtle temporal variations in accuracy. Therefore, a more detailed analysis of the dynamic of errors is required. To do so, an original approach borrowed from the cognitive control literature consists in the combined analysis of response accuracy and RTs using Conditional Accuracy Functions (CAFs) during a lexical decision task.

The CAFs display the probability of a correct response as a function of RTs, informing *per se* on the dynamic of errors over time. Three profiles have been identified (van Maanen et al., 2019): (1) a homogeneous distribution of errors across RTs, (2) slow errors, and (3) fast errors. The first profile refers to the independence of accuracy and RTs, implying that errors depend on factors unaffected by speed, such as attentional fluctuations and neural noise. The second profile corresponds to a higher probability of errors in the longest RTs, attributed to the urgency to respond before the response deadline implemented in RT tasks, even when decision processes are not fully completed (van Maanen et al., 2019). Finally, the last profile is characterized by a high probability of errors in the shortest RTs, commonly observed in cognitive control paradigms manipulating the stimulus-response congruency to create incongruent situations such as in the Simon task or the Stroop task. In these tasks, and especially in the incongruent trials, two competitive responses are co-activated: an automatic but not-required response (e.g., reading the word in the Stroop task), and a controlled and required response (e.g., naming the color in the Stroop task). Fast errors in incongruent trials are interpreted as a result of the unsuccessful inhibition of the automatic response (i.e., response capture, Ridderinkhof, 2002). Analyzing the distributions of lexical decision errors using CAF would thus help clarify whether word and pseudoword errors are contingent on automatic processes, controlled processes, or a combination of both.

Previous studies using CAF analyses during lexical decision reported different patterns of errors between words and pseudowords (e.g., Scaltritti et al., 2021; 2023), suggesting that errors do not occur for the same reason in the two conditions. However, results are mixed or insufficient to draw clear conclusions about exact CAF patterns for each condition. Regarding words, studies using CAF as an exploratory analysis did not report statistical analysis. Regarding pseudowords, while some CAF studies have shown fast errors (Fernández-López et al., 2022; Scaltritti et al., 2021; 2023), another reported fast errors specifically for the most wordlike pseudowords, such as transposed-letter pseudowords (e.g., “relovution” for the word “revolution”; Perea et al., 2023). Limitations that may explain these mixed findings include the repetition of items (e.g., Scalttriti et al., 2023) and the use of limited number of trials (Fernández-López et al., 2022; Perea et al., 2023; Scaltritti et al., 2021; 2023). The lack of convincing evidence probably stems from the fact that, to perform CAF analyses, the RT distribution has to be divided into small (five to seven) bins, requiring a consequent number of trials.

Given these methodological shortcomings, the current study aimed to enhance the analysis of the dynamic of errors in a lexical decision task using longer duration of stimulus display and a large number of non-repeated items. Additionally, since no previous research appears to have explored errors during visual word recognition and pseudoword categorization using both CAF analyses and the comparison of correct and error RTs, this study sought to address this gap. Based on previous literature, we expected to observe fast errors in pseudowords but did not make any specific hypothesis concerning the dynamic of word errors. Also, as a reading measure was collected as an exclusion variable and in line with previous findings (Horowitz-Kraus & Breznitz, 2011), exploratory analyses were conducted to examine the relationship between reading skills and lexical decision error dynamics.

## 2. Method

### 2.1. Participants

A total of 42 healthy volunteers were recruited at the University through advertisements on campus and social media (*M_age_* = 20.61, *SD_age_* = 2.54, 29 women). Inclusion criteria required that participants must have no history of neurological or psychiatric disorders, no reported learning disorders (e.g., developmental dyslexia), corrected-to-normal or normal vision, and be native French speakers. The study protocol was approved by the local ethics committee (2017-242-S55 on December 14^th^ of 2017). Participants provided their informed consent to participate in the study.

Nonverbal intelligence was assessed with the short version of the Raven’s Progressive Matrices (PM38, Raven & Court, 1998). Participants who scored below or in the 25^th^ percentile, suggesting lower non-verbal intelligence, were removed from statistical analysis (N = 2). Reading was assessed using the French “L’Alouette” test (Lefavrais, 1965) to exclude any participant with low reading skills. A reading efficiency score was calculated as \[
{\mathrm{CTL}}\;{\mathrm{ = }}\;{\textstyle{{WCR\;\;{\mathrm{ \times }}\;\;180} \over {rt}}}
\] , where WCR is the number of words correctly read among 265, and *rt* is the reading time (maximum time allowed = 180 seconds, Cavalli et al., 2018). The CTL was used as a threshold to discriminate between typical and atypical readers (e.g., a score below 402.2 is considered as atypical reading skills). Four participants had a CTL score below the cut-off. Therefore, out of the initial 42 participants, six were excluded from the statistical analysis, resulting in a remaining sample size of *N* = 36 (27 women). In our study, participants are presented with 500 words and 500 pseudowords, distributed across five bins for the CAF analyses (i.e., 100 observations in each combination). With a sample size of 36, we exceed Brysbaert and Stevens’ (2018) recommendation of at least 1600 observations per experimental condition, resulting in a total of 3600 observations in our design.

### 2.2. Lexical decision task

A total of 500 monosyllabic and bisyllabic words were selected from the French lexical database LEXIQUE 3 (New et al., 2001), and 500 paired pseudowords were created using letter replacement. All words were five to six letters long, with an average print lexical frequency of 27.95 per million. Pseudowords were created by replacing two to four letters in their paired words (e.g., the French word “achat” was used to create the pseudoword “achou”). Words and pseudowords were matched in terms of orthographic and phonological neighborhood, *t*(499) = –0.60, *p* = .548 and *t*(499) = 0.74, *p* = .460, respectively, as well as letter and bigram frequency, *t*(499) = 0.78, *p* = .433 and *t*(499) = 1.01, *p* = .313, respectively.

Half of the lexical decision trials were words, and the other half pseudowords with an equal distribution within each block (i.e., 100 words and 100 pseudowords). Each stimulus was presented only once to the participant.

The participant was placed in front of a screen to perform the lexical decision implemented in Matlab software and was asked to determine, as fast and as accurately as possible, whether the displayed stimulus was a real word or not by pressing the designated key on a standard AZERTY keyboard (i.e., D and K keys). The mapping rule was counterbalanced across participants. Each trial began with a fixation cross displayed for 400 ms, followed by the stimulus which remained on-screen until the participant responded. In the absence of a response, the stimulus disappeared after a delay of 1200 ms. The choice of a 1200 ms deadline aligns with the methodological standards of lexical decision tasks, providing a balance between speed and accuracy while maintaining a time pressure suitable for CAF analysis. The next trial began after 600 ms. A brief training phase of 20 trials was implemented to familiarize the participant with task instructions and the response device. During the training phase only, feedback was provided at the end of each trial for 800 ms (i.e., “*Well done, correct response*” - “Bravo, bonne réponse”, “*Incorrect response*” - “Réponse incorrecte” and “*Try to be faster*” - “Essayez d’être plus rapide” for an omission). The experimental phase of the lexical decision task was composed of five blocks of 200 trials each (1000 trials) without any feedback and lasted approximately 30 minutes.

The measures collected in the lexical decision task included reaction times (RTs in ms) and error rates (in %), both task-wide and condition-specific (i.e., word and pseudoword trials). Reaction times (ms) were also computed as a function of performance (Correct vs. Error) both task-wide and condition-specific.

### 2.3. Conditional Accuracy Function (CAF)

To investigate the dynamic of accuracy across RTs, CAFs were computed for each participant and each experimental condition (i.e., word vs. pseudoword). To achieve this, RTs were vincentized (Vincent, 1912) meaning that all RTs were sorted in ascending order and then divided into five bins of equal number of observations (i.e., quintiles). Five bins were selected to facilitate visual comparisons with previous CAF studies (e.g., Scaltritti et al., 2021; 2023) while ensuring the reliability of the measures based on 100 trials per experimental condition. We calculated accuracy for each quintile and plotted it as a function of the corresponding mean reaction times (RTs). The slope between the first and the second quintiles of the CAF was also calculated to assess the strength of lexical capture in the lexical decision task (Scaltritti et al., 2021), considered to be similar to the “response capture” observed in cognitive control tasks (i.e., the extent to which performance is influenced or disrupted by a dominant response tendency, van den Wildenberg et al., 2010).

### 2.4. Statistical analysis

The main goal of this research was to explore error distributions in a lexical decision task. To stabilize variance and improve the normality of error distributions, we applied an arcsine square root transformation to the raw accuracy values. Reaction times shorter than 150 ms or superior to 1200 ms were excluded from the analysis and a 2.5*standard deviation interval filter was calculated for each participant and used to eliminate potential interindividual performance outliers, resulting in the removal of 3% of trials within the whole sample.

First, differences in mean RTs were examined through an ANOVA with Performance (Correct vs. Error) and Condition (Word vs. Pseudoword) as two within-subject factors to test for a global error timing difference between the two experimental conditions. Second, the CAF pattern was analyzed through a two-way ANOVA on accuracy with Quintile and Condition as within-subject factors to specify the dynamics of errors. Finally, to explore the impact of reading skills, correlation analyses were conducted between the CTL score and the Correct-Incorrect RT difference in each experimental condition. Additionally, CAF patterns were analyzed as a function of reading groups, which were arbitrarily defined based on the median CTL score.

## 3. Results

### 3.1. Classical findings

Accuracy and reaction times in correct trials were analyzed as a function of Condition to verify the classical lexicality effects. Reaction times in correct pseudowords trials (*M* = 649.35 ms, *SD* = 49.46) were longer than RTs in correct words trials (*M* = 604.88 ms, *SD* = 41.71), *t*(35) = –9.40, *p* < .001, Cohen’s *d* = –0.94 (95% CI [–1.18, –0.70]). The accuracy was lower for words (*M* = 93.32%, SD = 2.69) than for pseudowords (*M* = 95.08%, SD = 2.89), *t*(35) = –4.03, *p* < .001, Cohen’s *d* = –0.70 (95% CI [–1.09, –0.31]).

### 3.2. Investigation of error and correct mean RTs

Table 1 summarizes the task-wide and condition-specific RTs as a function of the performance. The ANOVA results showed significant main effects of both Performance and Condition on RTs, *F*(1,35) = 4.40, *p* = 0.043, η_p_² = 0.11 and *F*(1,35) = 37.06, *p* < .001, η_p_² = 0.51, respectively. Also, the interaction effect between Performance and Condition was significant, *F*(1,35) = 17.90, *p* < .001, η_p_² = 0.34. Pairwise comparison indicated that RTs were significantly faster in error compared to correct trials in the pseudoword condition (*p* < .001). No significant RT difference was observed between error and correct trials within the word condition (*p* = .125).

**Table 1:** Means (and Standard Deviations) of Correct and Error Trials RTs.


VARIABLES	CORRECT TRIALS	ERROR TRIALS	*P*-VALUE

Task-wide	629.71 (51.18)	617.83 (63.95)	.043 *

Word trials	606.57 (41.44)	617.63 (60.09)	.125

Pseudoword trials	652.86 (49.95)	618.03 (68.45)	<.001 ***


*Note*. Significant differences are marked in bold. *: *p* < .050, ***: *p* < .001.

### 3.3. Conditional Accuracy Functions

**Figure 1:**
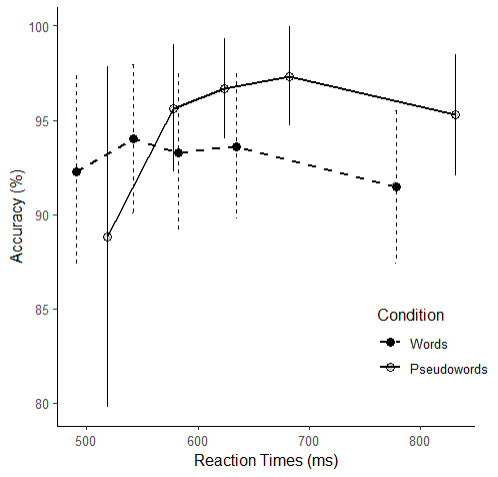
Conditional Accuracy Functions. *Note*. Each point represents the accuracy for one quintile and one condition. Error bars show standard deviations from mean accuracy.

The ANOVA revealed significant main effects of Quintile and Condition on accuracy, *F*(3, 96) = 22.07, *p* < .001, η_p_² = 0.39, and *F*(1, 35) = 23.11, *p* < .001, η_p_² = 0.40, respectively. The interaction effect Quintile x Condition was significant, *F*(3, 93) = 13.29, *p* < .001, η_p_² = 0.28, showing that error distribution across quintiles differed between the two conditions (Figure 1). The main effect of Quintile analyzed separately in the word and pseudoword conditions was significant in both conditions, *F*(2, 78) = 3.50, *p* = .031, η_p_² = 0.09 and *F*(3, 105) = 29.54, *p* < .001, η_p_² = 0.46, respectively.

Table 2 displays the values of accuracies as well as the differences between the accuracies as a function of Condition in each quintile. For the word condition, pairwise comparisons with Bonferroni correction indicated that the accuracy in Q5 was significantly lower than in Q2 and in Q4 (see Table 2, for the accuracy values). For the pseudoword condition, the accuracy in Q1 was significantly lower than accuracies in the fourth remaining quintiles (see Table 2 for the accuracy values). Also, accuracy in Q4 was significantly larger than in Q5. Table 3 summarizes the pairwise comparison of accuracy between quintiles for each condition separately.

**Table 2:** Means (and Standard Deviations) of Accuracy (%) as a function of Quintiles and Conditions.


CONDITIONS	Q1	Q2	Q3	Q4	Q5

Words	92.26 (5.09)	94.02 (3.94)	93.30 (4.18)	93.60 (3.84)	91.46 (4.06)

Pseudowords	88.81 (9.06)	95.63 (3.38)	96.67 (2.64)	97.33 (2.61)	95.30 (3.18)

*p*-value	*.056*	.010 *	<.001 ***	<.001 ***	<.001 ***


*Note*. Q refers to the quintiles. The *p*-values reflect the pairwise comparisons of accuracy between the two conditions in each quintile (Bonferroni correction was applied). Significant differences are marked in bold. *: *p* < .050, **: *p* < .010, ***: *p* < .001.

**Table 3:** Pairwise Comparisons of Accuracy Between Quintiles in each Condition.


PAIRED QUINTILES	WORD CONDITION		PSEUDOWORD CONDITION	
	
	*T*	*P*-VALUE	*T*	*P*-VALUE

Q1–Q2	–2.39	.224	**–6.80**	<.001 ***

Q1–Q3	–1.01	1.000	**–7.14**	<.001 ***

Q1–Q4	–1.00	1.000	**–8.97**	<.001 ***

Q1–Q5	1.08	1.000	**–4.74**	<.001 **

Q2–Q3	1.60	1.000	–1.781	.840

Q2–Q4	0.55	1.000	**–3.89**	.004 **

Q2–Q5	**3.04**	.045 *	0.82	1.000

Q3–Q4	–0.49	1.000	–1.50	1.000

Q3–Q5	2.84	.180	2.69	.107

Q4–Q5	**3.50**	.013 *	**4.21**	.002 **


*Note*. Significant differences are marked in bold. P-values were corrected using Bonferroni’s method. *: *p* < .050, **: *p* < .010, ***: *p* < .001.

Concerning the CAF slopes, the first one (Q1–Q2) in the word condition was significantly smaller (*M* = 0.03, *SD* = 0.07) than the one in the pseudoword condition (*M* = 0.11, *SD* = 0.11), *V* = 147, *p* = .006, Cohen’s *d* = 0.78. The last one (Q4–Q5) did not differ between the two conditions, *t*(35) = –0.42, *p* = .678, Cohen’s *d* = 0.09.

### 3.4. Exploratory analyses of the impact of reading skills in the dynamic of lexical decision’s errors

As reading skills were assessed, we explored their potential impact on the dynamics of lexical decision errors. To maximize the variability captured in CTL scores, we first opted for a correlation-based approach for this post-hoc data exploration. Based on Horowitz-Kraus & Breznitz (2011)’s approach, we calculated the difference between RTs in incorrect and correct trials in each condition for each participant (i.e., Correct RT - Incorrect RT). Thus, a positive RT difference would indicate that incorrect responses are faster than correct responses (i.e.,fast errors). Conversely, a negative RT difference indicates slower incorrect responses compared to correct responses (i.e., slow errors). Our results showed a marginal positive correlation between the CTL and the RTs difference in words, *r* = .33, *p* = .053. A more negative RTs difference in the word condition seems associated with lower CTL scores. However, no correlation was observed between the CTL and the RTs difference in the pseudoword condition, *r* = .04, *p* = .824. Comparison of CAF patterns according to reading level groups (i.e., groups created by median-split on CTL score) showed no significant results.

## 4. Discussion

The current study involved a detailed analysis of errors during visual word recognition (VWR), to provide new insights into the roots of incorrect print processing. To do so, we compared the dynamics of errors and correct responses in a lexical decision task (LDT) through classical mean RTs and a fine-grained analysis of Conditional Accuracy Functions (CAFs). We were unable to draw a specific hypothesis concerning the pattern of errors for words, but we expected to replicate a pattern of fast errors in pseudowords based on previous results (e.g., Fernández-López et al., 2022; Ratcliff et al., 2004; Scaltritti et al., 2021; 2023).

In accordance with our hypothesis for pseudowords, error RTs were overall faster than correct RTs, as observed in one experiment of Ratcliff et al. (2004). Using the CAF analysis, we observed that the accuracy in the first quintile was significantly lower than the accuracy of the other four quintiles, suggesting a fast error pattern, as observed in Scaltritti et al. (2021; 2023). Integrating visual word recognition into cognitive control models to interpret this finding, the pattern of fast errors in pseudowords suggests that lexical information is automatically activated during pseudoword processing. From the perspective of visual word recognition models, as proposed by Balota & Chumbley (1984), the strength of lexical activation depends on the proximity of the written string with a word (i.e., “wordness” in the diffusion model, Ratcliff et al., 2004). In such cases, the threshold for a “word” response is reached rapidly, increasing the likelihood of errors (Ratcliff et al., 2004). If not effectively inhibited, this activation interferes with the correct response, particularly in trials with the fastest RTs when inhibitory processes have insufficient time to be engaged (Ridderinkhof, 2002). Inhibitory processes are also integrated in some visual word recognition models, such as the leaky competing accumulator (LCA) model (Dufau et al., 2012) that postulates inhibitory interactions between competing responses. Initially, the automatic lexical activation facilitates the “yes” response while inhibiting the “no” response. The “no” response is facilitated when lexical activation has not reached the threshold in a certain amount of elapsed time, while progressively inhibiting the “yes’ response, thus explaining the increase in accuracy in the pseudoword condition in slower RTs. Regarding the relationship between fast pseudoword errors and reading skills, an exploratory analysis failed to reveal any effect of reading skills, as assessed using the “Alouette” reading test on the “lexical capture” (i.e., how much the precision decreases in Q1 compared to Q2). Future studies should explore how this “lexical capture” phenomenon is related to reading skills in larger and more variable samples (e.g., children during reading acquisition, dyslexic vs. typical readers). Also, it is worth noticing that this pattern of fast errors seems not to be observed for replaced-letter pseudowords, such as those used in the present study, when more difficult pseudowords are used in the lexical decision task (e.g., transposed-letters pseudowords in Perea et al., 2023). A pattern of fast errors is indeed expected to occur more for pseudowords that closely resemble real words (Ratcliff et al., 2004). Also, when transposed and replaced letter pseudowords are presented, the easiest condition of pseudowords may benefit from additional processing (e.g., deeper orthographic processing) to be successfully categorized, resulting in a reduced proportion of fast errors. Future studies are needed in order to describe the modulation of the fast error pattern by various factors such as reading skills, pseudoword difficulty or task instructions (e.g., see Romero-Ortells et al., 2024 for the impact of a delayed lexical decision task).

Regarding words, our results did not reveal any significant difference between error and correct RTs. This finding may seem to contradict Ratcliff et al. (2004)’s results. However, they reported specific error patterns according to word frequency: a pattern of fast errors for high frequency words and a pattern of slow errors for low frequency words. The material of the present study comprised words distributed according to a large range of lexical frequencies (i.e., from 1.08 to 153.31 per million), which might explain the lack of specific error pattern found. This result is consistent with the global CAF pattern, revealing no significant difference between the accuracies in the first and last quintiles, suggesting a globally homogeneous distribution of errors across RTs. However, some slight significant differences were observed between the last two quintiles, suggesting slow word errors. The last slopes did not differ between words and pseudowords, suggesting that these slow errors are unrelated to the experimental conditions. In the cognitive control framework, slow error patterns are interpreted as the result of the implementation of a response deadline (Van Maanen et al., 2019) that can be found as an individual decision-making criterion in the Multiple Read-Out model (MROM, Grainger and Jacobs, 1996). However, based on visual word recognition models, other factors can be taken into account to understand these slow errors. Within drift diffusion models, slow errors in both conditions might be explained by trial-to-trial adjustments of drift rates (Ratcliff, 1978). The leaking accumulator model proposes trial-by-trial adjustments of the response threshold according to the accuracy of the previous trial (Dufau et al., 2012). Finally, slow errors are mostly observed in situations where the accuracy is emphasized and/or within difficult tasks (“evidence-quality” errors, Damaso et al., 2020). Future studies could thus explore the effect of word parameters (e.g., lexical frequency) and reading skills using balanced material, to test if a higher difficulty within the lexical decision task, either manipulated by word frequency and/or reading skills differences, might strengthen the slow error pattern in the word condition. Slow errors, as observed in longer incorrect RTs in erroneous trials compared to correct trials, were indeed reported in low frequency words (Ratcliff et al., 2004) and more strongly in dyslexic readers than in typical readers (Horowitz-Kraus & Breznitz, 2011). In our study, while no effect of reading skills was observed on the global CAF pattern in the word condition, a close-to-significant correlation was reported between reading efficiency and the RT difference between correct and incorrect word trials. This result might suggest that the poorer the reading skills in our sample, the slower were the errors compared to correct trials in the word condition, and is consistent with the findings of Horowitz-Kraus et Breznitz (2011) in dyslexic readers. These findings may indicate that slow errors in word trials are characteristic of poor reading skills. For poor readers, some words would not generate enough lexical activation to reach the word response criterion (e.g., Grainger & Jacobs, 1996) or to sufficiently inhibit the “no” response (e.g., Dufau et al., 2012). In these cases, a decision-making criterion would intervene such as after a certain time (i.e., decision deadline), participants would assume that the written string does not exist and make a “no” response (Balota & Chumbley, 1984; Grainger & Jacobs, 1996) leading to the observed slow errors.

## 5. Conclusion

By detailing the analysis of correct and error RTs and using CAF analysis, the present study investigated the error dynamics in lexical decision among typical adult readers. First, we confirmed the pattern of fast errors in pseudoword trials. Drawing on visual word recognition models and cognitive control literature, this finding suggests that pseudoword errors are linked to uninhibited automatic lexical activation, which disrupts the execution of the correct response in the fastest RTs. Concerning words, findings revealed that errors were globally independent of RTs. Still, slow errors were observed in both words and pseudowords. According to the literature, slow errors could depend on various factors such as a deadline, or trial-by-trial adjustments and may be more sensitive to differences in reading skills and stimulus difficulty. Further investigation is needed to specify the dynamic of errors while accounting for additional factors such as reading skills. Overall, this study clarifies the error distribution pattern in a lexical decision task, and offers new insights into the origins of errors, suggesting future research directions.

## Data Accessibility Statement

The data that support the findings of this study are available on the *Recherche Data Gouv* platform (https://doi.org/10.57745/VZ8HOR). The dataset is accompanied by relevant metadata to ensure reproducibility and transparency.
